# Pregnancy Following Imperfect Coitus; Unyielding Hymen

**Published:** 1896-12

**Authors:** G. A. Himmelsbach

**Affiliations:** Assistant attending physician Buffalo General Hospital, 137 W. Tupper Street


### Clinical Report.

### PREGNANCY FOLLOWING IMPERFECT COITUS; UNYIELDING HYMEN.

By G. A. HIMMELSBACH, M. D.,
Assistant attending physician Buffalo General Hospital.

MRS. ——— ; age, 34 ; married on Wednesday, November 25, 1895. On the Sunday preceding Wednesday she ceased menstruating (*i. e.*, three days before). Marital relations had not proceeded far before it was discovered that sexual intercourse was impossible on account of a tight (small) and unyielding condition of the hymen. The couple were now on their honeymoon, and naturally this condition of affairs proved a great disappointment to them both. They, however, decided not to consult a physician until their return home. During their absence, and in fact until near Christmas, *i. e.*, one month following their marriage, they did not cease their efforts at natural intercourse, but all was in vain. At last discouraged with their futile attempts, Mrs. ——— consulted an able physician here, who after his examination remarked "that he did not wonder at their repeated failure." He inquired of patient "whether she had ever menstruated," presumably considering the case one of imperforate hymen, and her reply was in the affirmative and regularly since puberty. This, I am told, was a surprise to him, because, as he remarked, "he did not see how the menstrual flow found its escape." At her request he made several incisions, increasing the lumen of the vaginal entrance; for a week or so subsequent to this, coitus was interdicted, and when it was permitted was extremely painful.

On the morning of June 24th, I was hastily summoned to the residence of the young woman, and on my arrival learned that thirty-six hours before, while walking leisurely on the street, she suddenly lost all control of her urine. She hastened home and found, much to her surprise, that instead of the flow of urine coming to a stop it continued to dribble. She soon was taken with cramp-like pains, but even this did not alarm her. She was satisfied that they could not be labor pains, for she did not expect her confinement till the latter part of August or September 1st, *i. e.*, in two months at the very earliest.

When I first saw her, examination showed the cervix to be entirely obliterated and the external os the size of a silver dollar, vertex presenting, and, much to her surprise, I informed her of the inevitable result, and at 10 A. M. I delivered her of a male child ; first position, cord about the neck ; the child soon cried and showed other evidences of life, and after a long wait the cord was tied and child wrapped in woolens and carried to the hearth. I did not permit him to be handled and allowed him to remain unwashed for several hours, when I considered his condition warranted careful handling with safety.

The child's head was well covered with dark hair ; its finger and toe nails were fairly well grown, pulse 144, a well-formed head and body, but very small, weighing 3¾ pounds ; in all, appearing to me like a child of about seven months.

The last two hours of labor was exceedingly trying and painful, owing to the atresia of the vulval outlet. After complete cervical dilatation the head descended and remained on the perineum for two hours, giving time for the perineal fibers to arrange themselves ; but in spite of this delay a slight median laceration occurred and was immediately sutured.

I relate this history in detail because of its extreme interest and unusual occurrence. I desire principally to show that conception can take place without a natural coitus. It will be observed that from the day of marriage to the day of birth was exactly seven months lacking one day (November 25th to June 24th). It will be remembered also that she ceased menstruating three days before her marriage, so I assume that conception must have taken place the first few days of marital life, notwithstanding the fact that natural coition was not had until early in January, over a week after the operation.

137 W. Tupper Street.

